# Lovastatin reversed the enhanced sphingomyelin caused by 27-hydroxycholesterol in cultured vascular endothelial cells

**DOI:** 10.1016/j.bbrep.2015.11.024

**Published:** 2015-12-03

**Authors:** Qi Zhou, Allan Luo, Fred A Kummerow

**Affiliations:** a Department of Comparative Bioscience, University of Illinois, 205 Burnsides Research Laboratory, 1208 W. Pennsylvania Avenue, Urbana, IL 61801, USA; b Huazhong University of Science and Technology, 1037 Luoyu Road, Wuhan, China

**Keywords:** Lovastatin, Phosphatidylcholine, Sphingomyelin, 27-hydroxycholesterol CTP: phosphocholine cytidylyltransferase

## Abstract

Statins have pleiotropic properties which are involved in inhibiting the thrombogenic response. In this study, the effects of lovastatin on two phospholipids, phosphatidylcholine and sphingomyelin, were studied in cultured endothelial cells in the presence of an oxysterol, 27-hydroxycholesterol. After the cells were cultured with 50 nM of lovastatin for 60 h, lovastatin was found to decrease the incorporation of [^3^H]choline into phosphatidylcholine and sphingomyelin, inhibited CTP: phosphocholine cytidylyltransferase (CT) activity without altering the activity of sphingomyelin synthase and neutral sphingomyelinase. And lovastatin was not found to have a direct inhibitive effect on activity of CT. Exogenous mevalonic acid or cholesterol reversed the reduction of cholesterol concentration that was caused by lovastatin, but had no significant effect on the diminished [^3^H]sphingomyelin by lovastatin. The increase of [^3^H]sphingomyelin by 27-hydroxycholesterol was not detected in the presence of lovastatin. These findings suggest that (1) lovastatin can reduce sphingomyelin content by means of inhibiting phosphatidylcholine synthesis; and (2) The decrease in sphingomyelin is not related to the diminished cholesterol concentration or mevalonate-derived intermediates. This inhibitive effect of lovastatin on sphingomyelin may benefit cellular calcification caused by sphingomyelin.

## Highlights

• Lovastatin decreased phosphatidylcholine concentration by inhibiting CT activity.
• Decreased sphingomyelin by lovastatin came from inhibited phosphatidylcholine.
• Decreased sphingomyelin by lovastatin was not related to diminished cholesterol.

## 1 Introduction

Oxysterols, present in human serum and lesions, have been linked to the initiation and progression of atherogenesis [1], [2]. 27-Hydroxycholesterol, one type of oxysterols, is synthesized in the liver by CYP27A1 as the first intermediates of classic and acidic bile acid synthetic pathways [3] and by non-hepatic cells [4]. It has been detected in mammalian tissues at very low concentration [5] and was enriched in pathologic structures such as atherosclerotic lesions [1], [5]. The plasma from patients who had coronary artery bypass surgery had a higher concentration of 27-hydroxycholesterol than that in the plasma from age and sex matched controls [5]. One of the atherogenic effects of oxysterol was to enhance sphingomyelin concentration [6]. It is, therefore, not surprising to observe an increase of both oxysterols and sphingomyelin in atherosclerotic plaque [1], [5], [7]. And sphingomyelin was also found to result in a cellular calcium accumulation [6], [8] because of its location on the exterior of the plasma membrane [9]; and its negative charge is accessible to ionic bonding with Ca^++^ [10].

Hydroxymethylglutaryl-coenzyme A (HMG-CoA) reductase is a rate-limiting enzyme in the mevalonate pathway. Its inhibitors, known as statins, have anti-atherogenic effects through reductions in circulating LDL cholesterol. Besides the reducing effect on *de novo* synthesis of cholesterol through blocking HMG-CoA [11], statins also showed to have pleiotropic effects [12], [13], including the reduction of phospholipids [14]. And the reduced phospholipids by statins were found independent of their decreasing effect on cholesterol [15], [16]; although the decreased mevalonate-derived intermediates were also related to this effect of statins [14]. A reduced proportion of sphingomyelin and phosphatidylcholine was detected in the plasma of the patients who took high-dose (80 mg) simvastatin for 6 weeks [17]. Simvastatin was also observed to reduce lysophosphatidylcholine content in LDL by directly inhibiting lipoprotein-associated phospholipase A_2_ [16]. However, there are some inconsistent findings [18], [19], [20], [21]. One study showed that atorvastatin significantly increased phospholipid content in erythrocyte membrane of guinea pigs [18]; but another study indicated that atorvastatin reduced the plasma concentration of phosphatidylcholine in humans [19]. Simvastatin was once found to raise liver CTP: phosphocholine cytidylyltransferase (CT) activity in normolipidemic rats [20], but on another occasion it was not observed to affect this enzyme's activity in normolipidemic rabbits [21]. The present study is our further effort in investigating the pleiotropic effect of lovastatin [13]. Our focus this time is on whether lovastatin could reduce sphingomyelin enhanced by 27-hydroxycholesterol on cultured vascular endothelial cells, which are in direct contact with toxicants in the circulation, and are also involved in atherosclerotic plaque formation.

## 2 Materials and methods

### 2.1 Materials

[1,2-^14^C]acetate was obtained from NEN Research Products (Boston, MA, USA) and [methyl-^3^H]choline chloride, [methyl-^14^C]phosphorylcholine, [methyl-^14^C]sphingomyelin, and phosphatidyl[N-methyl-^3^H]choline were purchased from American Radiolabeled Chemicals, Inc. (St. Louis, MO, USA). 27-Hydroxycholesterol was purchased from Research Plus Inc. (Manasquan, NJ, USA). Lovastatin, mevalonic acid, cholesterol, fetal bovine serum (FBS), Eagle's minimum essential medium (MEM), lipoprotein deficient serum (LDS), and other reagents were purchased from Sigma (St. Louis, MO, USA).

The inactive lactone form of lovastatin was converted to the active form by dissolving 4 mg of lovastatin in 0.1 mL of absolute ethanol. Then 0.15 mL of 0.1 N NaOH was added. After heating at 50 °C for 2 h, the resulting solution was neutralized with HCl to a pH of approximately 7.2 and brought up to a volume of 1 mL with distilled water (Merck Research Laboratories). Stock solution (10 mM) was stored at −20 °C. The cells were exposed to lovastatin at levels from 5 to 200 nM at designed periods. 27-Hydroxycholesterol was dissolved in absolute ethanol at a concentration of 25 mM and stored at −20 °C. Prior to use, 1 μL of 27-hydroxycholesterol in ethanol was first dispersed in 20 μL of 0.05% bovine serum albumin and then transferred into the culture medium. 27-Hydroxycholesterol was used at levels of 1.25 and 12.5 μM. The concentrations of [^3^H]choline and [^14^C]acetate were used at levels of 1 and 2 μCi/mL of culture medium, respectively.

### 2.2 Cell culture and treatment

Human endothelial cells from umbilical veins were obtained from American Type Culture Collection (Manassas, VA, USA) and the passages of the cells used in this study varied from 5 to 13. The cells were cultured in MEM supplemented with 10% FBS in a 5% CO_2_ incubator at 37 °C. When the cells in 25 or 75 cm^2^ flasks (Corning Life Sciences, Lowell, MA, USA) had grown to 50% confluences, they were cultured with lovastatin and/or 27-hydroxycholesterol in MEM containing 5% LDS for the designed periods. In the study of cholesterol and phosphatidylcholine synthesis, [^14^C]acetate or [^3^H]choline was added into the dishes prior to 4 h of culturing with lovastatin.

### 2.3 Cholesterol concentration and syntheses

For measuring cholesterol concentration, the cellular lipids were extracted [8] after aliquots were taken for protein assay (Bio-Rad, Hercules, CA, USA). The lipid extracts were subjected to thin-layer chromatography on silica gel G plates using hexane: diethyl ether: acetic acid (85:15:2, v:v:v). Following identification with iodine, the spots of free cholesterol band on plates were quantitatively collected, respectively. The concentrations of cholesterol were measured [22].

For measuring cholesterol synthesis, the lipid extracts from [^14^C]acetate-treated cells were subjected to thin-layer chromatography as mentioned above. The spots of free cholesterol on plates were collected and radioactivities were counted. The nonspecific activities obtained from some silica gel on blank spaces were subtracted from sample radioactivity.

### 2.4 Phosphatidylcholine and sphingomyelin syntheses and sphingomyelin concentration

For measuring incorporation of [^3^H]choline into phosphatidylcholine and sphingomyelin, lipid from [^3^H]choline-treated cells were extracted as mentioned above and were subjected to thin-layer chromatography on silica gel G plates using chloroform: methanol: acetic acid: water (25:15:4:2, v:v:v:v) [23]. The spots of phosphatidylcholine and sphingomyelin on the plates were collected and radioactivities were counted. For determining concentration of sphingomyelin, the lipid extracts were subjected to thin-layer chromatography as mentioned above. The spots of sphingomyelin on the plates were collected and the phosphorus (Pi) in each sample was then determined.

For measuring whether the inhibited sphingomyelin synthesis by lovastatin resulted from the reduced cholesterol, 10 μM of mevalonic acid or 25 μM of cholesterol was used at the same time lovastatin was added into the flasks. The concentrations of cholesterol and labeled sphingomyelin were determined after 60 h of the culturing.

### 2.5 Enzyme activities

For measuring the activity of CT, the cells were washed twice with normal saline, scraped, and homogenized in 200 μL of homogenization buffer [24]. Homogenates were sonicated in a water bath sonicator for 20 min and centrifuged (1000×g) to remove cell debris. After 20 μg of protein in supernatant were mixed with assay buffer containing 50000 cpm of [^14^C]phosphorylcholine [24] in a final volume of 50 μL, the mixed samples were cultured at 37 °C for 1 h. To stop the reaction, the tubes were immersed in a boiling water bath for 2 min. Next, 40 μl of each sample was spotted onto preadsorbent Silica Gel G thin-layer plates and developed in 95% ethanol: 2% NH_4_OH (1:1, v:v) [25]. The spots of CDP-choline on plates were collected. Radioactivities were counted and calculated. To investigate whether lovastatin has a direct inhibit effect on CT, supernatants from non-treated cells were exposed to lovastatin for 5 min before assay buffer was added. Then the procedure was as performed the same way as mentioned above.

For determining the activity of phosphatidylcholine: ceramide phosphocholinetransferase (sphingomyelin synthase), the method of Vivekananda et al. [26] was used with the following modifications. In brief, the cells were washed with ice-cold Hanks Balanced Salt Solution, harvested, lysed in hypotonic cold 1 mM MgCl_2_ and homogenized with a Dounce homogenizer. The homogenate was centrifuged at 1000×*g* for 5 min and 50 μg of protein in supernatant were added into an assay buffer containing 10 mM HEPES (pH 7.4), 3 mM MnCl_2_, 1% fatty acid-free bovine serum albumin and 0.05 μCi of phosphatidyl[^3^H]choline in a total volume of 100 μL. The reaction mixture was cultured for 6 h in a shaking water bath at 37 °C, terminated by addition of 1 ml of 0.2 M methanolic NaOH and then immediately followed by the addition of 0.5 mL of 0.45 M HCl. The lower organic phase containing sphingomyelin was separated by thin-layer chromatography and radioactivities were counted.

For assaying the activity of neutral sphingomyelinase (NSMase), the treated cells were scraped from the flasks, harvested by centrifugation, re-suspended in hypotonic buffer containing 1 mM NaCO_3_, 2 mM CaCl_2_, 1 mM NaHSO_3_, 1 mM benzamidine and 0.1 mM phenylmethylsulfonyl fluoride. After the cells were sonicated for 20 s in ice water, the resultant homogenate (20 μg of protein) was used for the assays of activity of SMase [27]. Enzymatic activity of SMase was measured by the formation of radioactive phosphocholine from [N-methyl-^14^C]sphingomyelin. The assay mixture for NSMase contained 0.1 M Tris/HCl, pH 7.0, 1 mM dithiothreitol, 10 mM MgCl_2_, 0.05% Triton X-100, 1.2 M KCl, 22 μM [Nmethyl-^14^C]sphingomyelin (20,000 cpm), and 20 μg protein of cell homogenate in a total volume of 50 μL. After incubation for 30 min at 37 °C, reactions were terminated by the addition of 4 volumes of chloroform/methanol (2:1, v:v). The radioactivity of phosphocholine recovered from the upper aqueous layer was determined in a liquid scintillation counter.

### 2.6 Cytotoxicity

The effects of lovastatin and/or 27-hydroxycholesterol on cell viability were determined by the 3-(4,5-Dimethyl-2-thiazolyl)-2,5-diphenyl-2H-tetrazolium bromide (MTT) assay. In brief, after the cells in 24-well plates were exposed to lovastatin and/or 27-hydroxycholesterol, the medium was removed and the cells were incubated in 540 μL of MEM and 60 μL of 2.5 mg/ml MTT solution for 4 h at 37 °C. Afterward, the medium with MTT was removed and 300 μL of dimethyl sulfoxide (DMSO) was added to each well and plates were shaken for 10 min. Absorbance was read at 570 nm [28]. Viability was expressed as a percentage of absorbance of treated cells to untreated cells (control).

### 2.7 Statistical analysis

Data were subjected to ANOVA and a Student–Newman–Keuls method. Differences with *P*<0.05 were considered significant. All data are presented as mean±standard error (S.E.M.).

## 3 Results

**Fig. 1:**
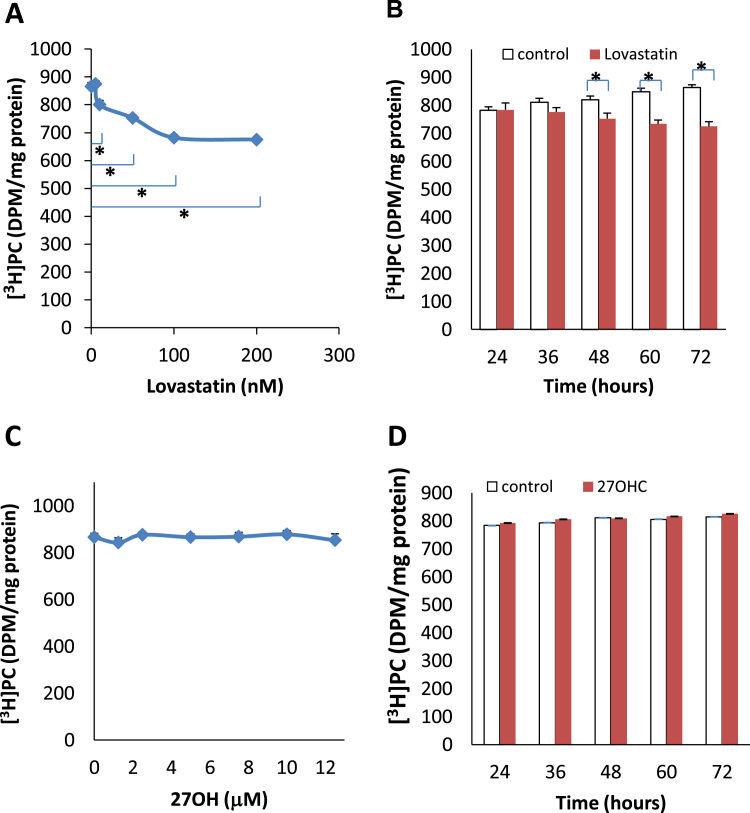
Incorporation of [^3^H]choline into phosphatidylcholine (PC) in the cultured endothelial cells treated with (A) levels of lovastatin from 5 to 200 nM for 48 h (B) 50 nM of Lovastatin from 24 to 60 h (C) levels of 27-hydroxycholesterol (27OH) from 1.25 to 12.5 mM for 48 h and (D) 2 mM of 27OH from 24 to 60 h in the presence of lipoprotein deficient serum in the culture medium. Results are expressed as mean±S.E.M. of duplicate for each independent determination in eight experiments. *P<0.05.

To prevent the cells from obtaining cholesterol from the culture medium, the cells were exposed to lovastatin in lipoprotein-free medium. Lovastatin induced a dose-dependent decrease in the incorporation of [^3^H]choline into phosphatidylcholine after 48 h of the incubation (Fig. 1A). The incorporation was decreased from 877±16 DPM/mg protein (control) to 675±11 DPM/mg protein (200 nM lovastatin). This significant decrease started at a concentration of 10 nM lovastatin. The inhibited incorporation not only occurred with the enhanced dose of lovastatin, but also took place when incubating time was extended. A significant inhibition of incorporation of [^3^H]choline (752±20 DPM/mg protein) was observed after 48 h in the cells cultured in a medium containing 50 nM of lovastatin, compared with the control (820±14 DPM/mg protein) (Fig. 1B). However, 27-hydroxycholesterol, with the chosen concentrations (Fig. 1C) and the designed incubation periods (Fig. 1D), had no effect on the incorporation of [^3^H]choline into phosphatidylcholine.

**Fig. 2:**
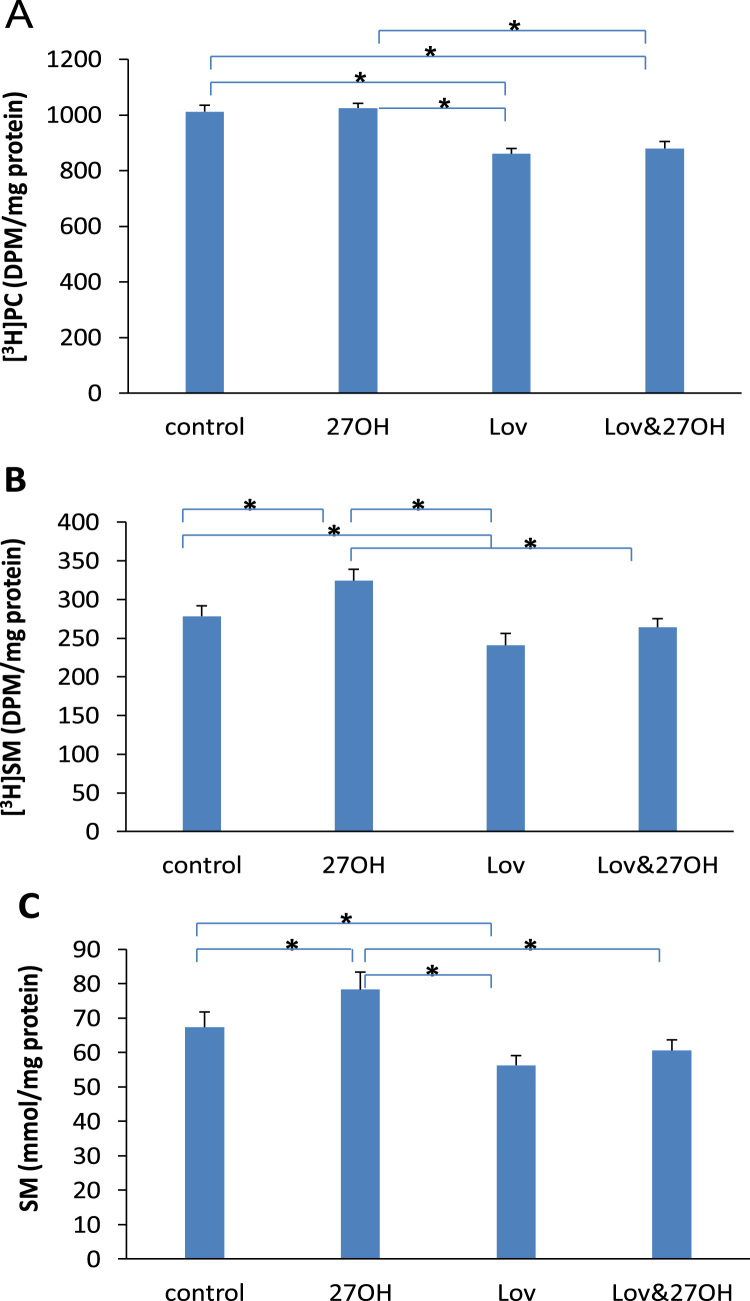
Incorporation of [^3^H]choline into phosphatidylcholine (PC) (A) and sphingomyelin (SM) (B) and the concentration of SM (C) in the cultured endothelial cells treated with 50 nM of lovastatin (Lov) and/or 2 μM of 27-hydroxycholesterol (27OH) for 60 h. Results are expressed as mean±S.E.M. of duplicate for each independent determination in eight experiments. *P<0.05.

The incorporation of [^3^H]choline into phosphatidylcholine and sphingomyelin as well as the concentration of sphingomyelin were altered by administration of lovastatin and 27-hydroxycholesterol as shown in Fig. 2. Fig. 2A showed that lovastatin inhibited the incorporation of [^3^H]choline into phosphatidylcholine. The incorporation decreased from 1012±23 (control) to 860±19 DPM/mg protein (lovastatin). This decrease was not affected by the presence of 27-hydroxycholesterol. The incorporation was at 880±25 DPM/mg protein in both lovastatin- and 27-hydroxycholesterol-treated cells. Lovastatin decreased both incorporation of [^3^H]choline into sphingomyelin (from 279±13 in untreated cells to 241±15 DPM/mg protein in treated cells) (Fig. 2B) and sphingomyelin concentration (from 67±4.4 in untreated cells to 56±2.8 mmol/mg protein in treated cells) (Fig. 2C). In contrast, 27-hydroxycholesterol increased significantly the incorporation to 325±16 DPM/mg protein and the concentration to 78.3±5.1 mmol/mg protein. After the addition of lovastatin into 27-hydroxycholesterol-treated cells, the stimulated incorporation and sphingomyelin concentration caused by 27-hydroxycholesterol returned to 264±11 DPM/mg protein and 60.1±3.1 mmol/mg protein, respectively.

**Fig. 3:**
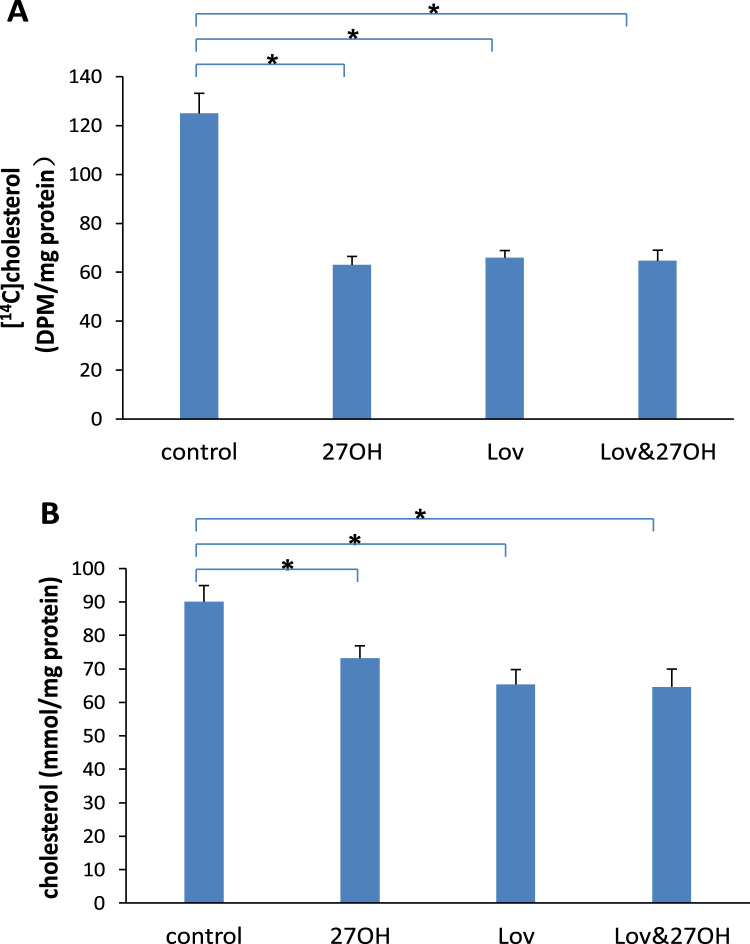
Incorporation of [^14^C]acetate into cholesterol (A) and the concentration of cholesterol (B) in the cultured endothelial cells treated with 50 nM of lovastatin (Lov) and/or 2 μM of 27-hydroxycholesterol (27OH) for 60 h. Results are expressed as mean±S.E.M. of duplicate for each independent determination in eight experiments. *P<0.05.

The amounts of newly synthesized [^14^C]cholesterol in both lovastatin- (66±2.9 DPM/mg protein) and 27-hydroxycholesterol-treated (63±3.5 DPM/mg protein) cells were decreased, compared with control (125±8.2 DPM/mg protein) (Fig. 3A). Similar changes were observed in cholesterol concentration (Fig. 3B). The concentration decreased significantly to 65.4±4.4 mmol/mg protein in lovastatin-treated cells and 73.2±3.7 mmol/mg protein in 27-hydroxycholesterol-treated cells, compared of 90.1±4.8 mmol/mg protein in control. When lovastatin and 27-hydroxycholesterol were added together, no further decrease of either newly synthesized [^14^C]cholesterol or cholesterol concentration was observed. These results suggested a similar mechanism of inhibitive effects on cholesterol synthesis by lovastatin and 27-hydroxycholesterol.

**Fig. 4:**
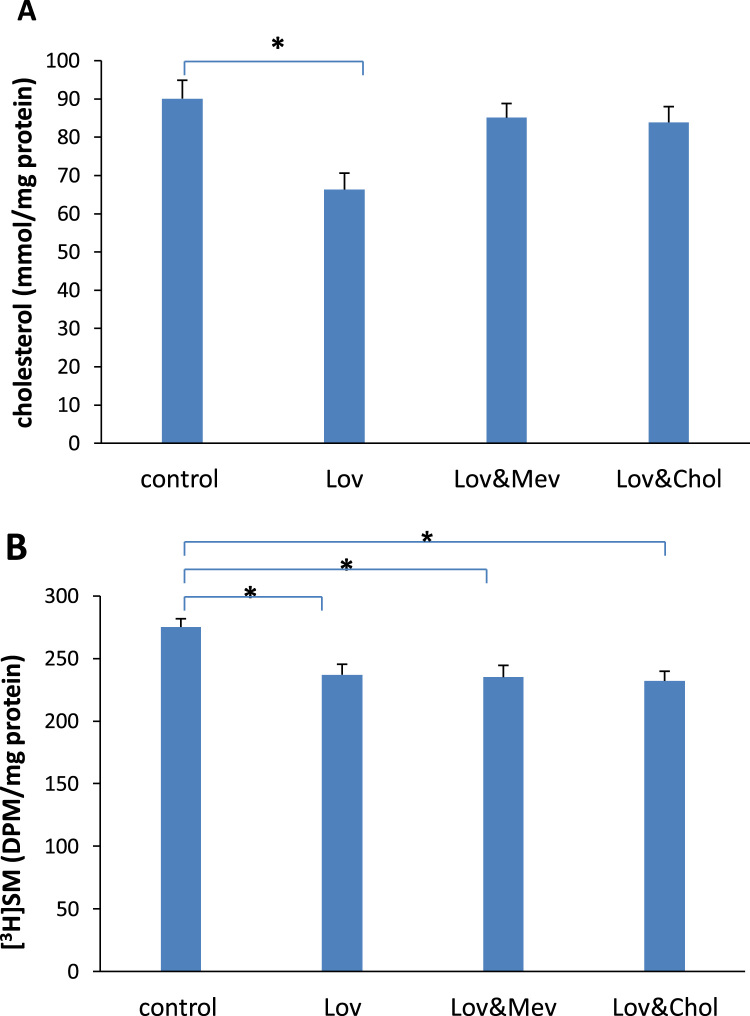
The concentration of cholesterol (A) and incorporation of [^3^H]choline into sphingomyelin (SM) (B) in the cultured endothelial cells treated with 50 nM of lovastatin (Lov) alone and with 10 μM of mevalonic acid (Mev) or 25 μM of cholesterol (Chol) for 60 h. Results are expressed as mean±S.E.M. of duplicate for each independent determination in eight experiments. *P<0.05.

To eliminate a possible inhibitive effect of cholesterol or mevalonic acid on the incorporated [^3^H]choline into sphingomyelin, mevalonic acid or cholesterol was added to the medium containing lovastatin. Fig. 4A shows that the decreased concentration (from 91.1±4.8 to 66.3±4.3 mmol/mg protein) of cholesterol caused by lovastatin was significantly reversed by the addition of cholesterol (83.9±4.1 mmol/mg protein) or mevalonic acid (85.1±3.7 mmol/mg protein). The inhibited incorporation of [^3^H]choline into sphingomyelin, however, was not obviously altered (Fig. 4B).

**Fig. 5:**
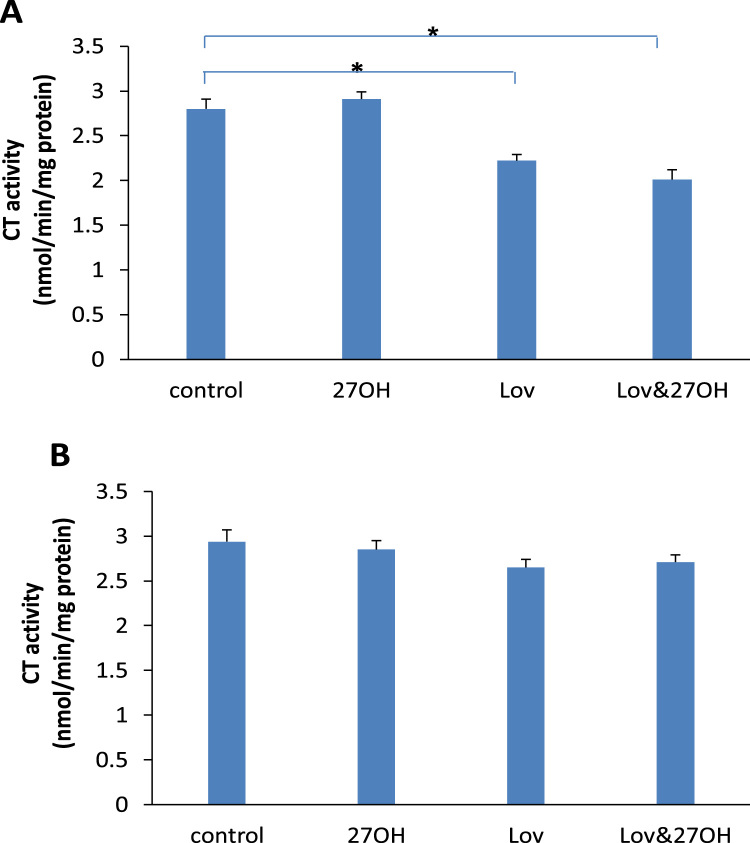
The activities of CTP:phosphocholine cytidylyltransferase (CT) in the cultured endothelial cells (A) and in supernatant (B) treated with 50 nM of lovastatin (Lov) and/or 2 μM of 27-hydroxycholesterol (27OH) for 60 h and 1 h respectively. Results are expressed as mean±S.E.M. of duplicate for each independent determination in eight experiments.,*P<0.05.

When the cells were exposed to lovastatin, it caused a significant reduction of the activity of CT. The CT activity was decreased to 2.22±0.07 (alone) and 2.01±0.11 (with 27-hydroxycholesterol) from 2.85±0.09 (control), respectively (Fig. 5A). However, this inhibiting effect of lovastatin on the activity of CT was not detected when it was added into the supernatant (Fig. 5B). 27-hydroxycholesterol had no effect on the activity of CT (Fig. 5).

**Fig. 6:**
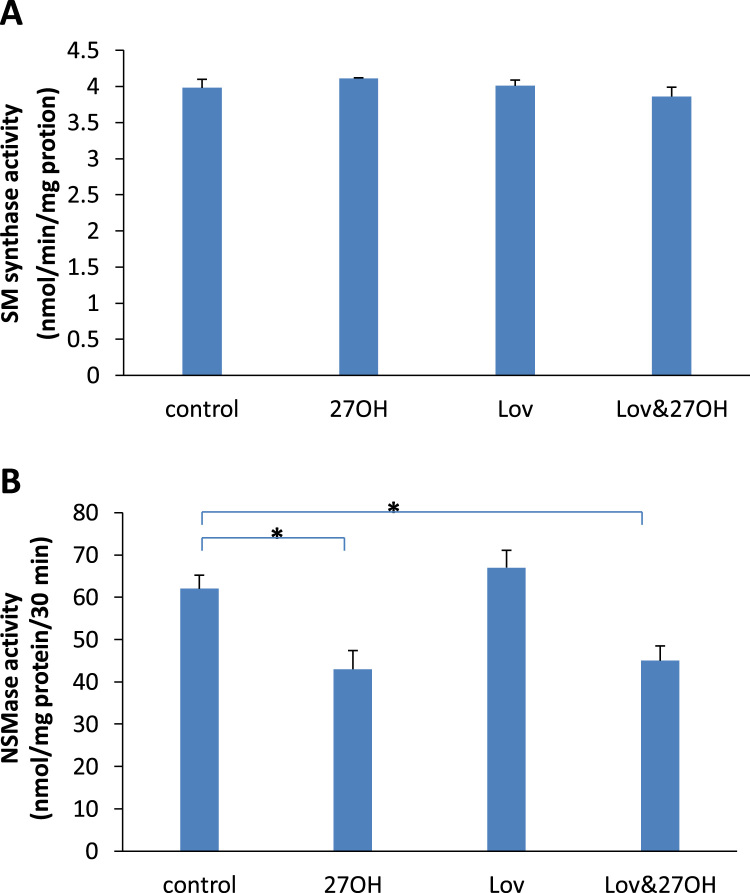
The activities of sphingomyelin (SM) synthase (A) and neutral sphingomyelinase (NSMase) (B) in the cultured endothelial cells treated with 50 nM of lovastatin (Lov) and/or 2 μM of 27-hydroxycholesterol (27OH) for 60 h. Results are expressed as mean±S.E.M. of duplicate for each independent determination in eight experiments. *P<0.05.

The effect of lovastatin on sphingomyelin synthase and NSMase was shown in Fig. 6. Lovastatin had no effect on the activities of sphingomyelin synthase (Fig. 6A) and NSMase (Fig. 6B) during the experimental periods. However, the activity of NSMase was significantly inhibited by 27-hydroxycholesterol, from 62±3.2 (control) to 43±4.1 (alone) and 45±3.5 (with lovastatin), respectively (Fig. 6B). The activity of sphingomyelin synthase was not affected by 27-hydroxycholesterol (Fig. 6A).

**Fig. 7:**
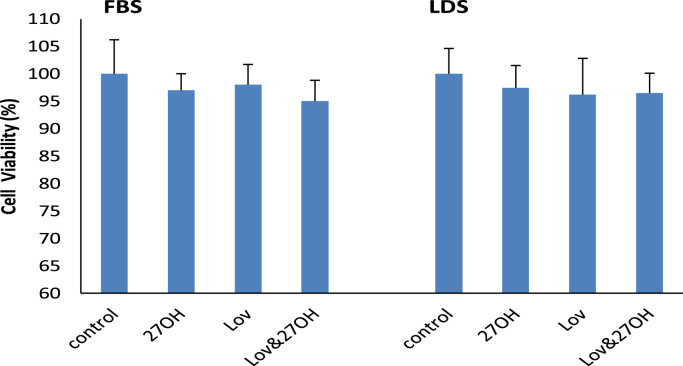
Effect of 50 nM of lovastatin (Lov) and/or 2 μM of 27-hydroxycholesterol (27OH) on cell viability of the cultured endothelial cells after 60 h of the incubation in the presence of fetal bovine serum (FBS) or lipoprotein deficient serum (LDS) in the culture medium, respectively. Results are expressed as mean±S.E.M. of duplicate for each independent determination in four experiments.

No significant difference in cell cytotoxicity was observed either in the lovastatin- and/or 27-hydroxycholesterol-treated cells, or in medium containing FBS or LDS (Fig. 7). The visible cells in lovastatin- and/or 27-hydroxycholesterol-treated cells were more than 95%, compared to control.

## 4 Discussion

In this study, we have shown that an increased sphingomyelin caused by 27-hydroxycholesterol was significantly reversed by lovastatin.

It is known that phosphatidylcholine is a precursor for the synthesis of sphingomyelin [29]. And sphingomyelin is synthesized mainly by the phosphorylcholine headgroup's being directly transferred from phosphatidylcholine to ceramide through sphingomyelin synthase. We therefore first studied the effect of lovastatin on phosphatidylcholine. We found that lovastatin significantly reduced, at both dosages and time points, the incorporation of [^3^H]choline into phosphatidylcholine. This indicates that lovastatin had an inhibitive effect on phosphatidylcholine synthesis in cultured vascular cells. Since phosphatidylcholine synthesis takes place through the “CDP-choline” pathway where CT is the rate-limiting enzyme, we measured the activity of this enzyme. We found that CT activity was inhibited by lovastatin. This indicates that the inhibitive effect of lovastatin occurred on the *de novo* synthesis of phosphatidylcholine. This finding was supported by a previous report that simvastatin, another HMG-CoA reductase inhibitor with a similar chemical structure as lovastatin, lowered activity of CT in HepG2 cells [30]. Since we did not observe a dicreased CT activity by adding lovastatin into the supernatant, we suggest that the inhibiting effect of lovastatin on CT was indirect, and the mechanism needs to be further studied.

The data that lovastatin treatment resulted in a decrease of sphingomyelin, but did not change the activities of sphingomyelin synthase and NSMase shows that the reduced sphingomyelin was not from the alteration of either sphingomyelin synthesis or its metabolism in the presence of lovastatin, but was from the diminished phosphatidylcholine caused by lovastatin.

Sphingomyelinase (SMase) is known to classify into two major groups, a lysosomal SMase called acid SMase (ASMase) and a cell membrane-associated Mg^++^-active SMase termed NSMase [31]. NSMase contributes to the catabolism of sphingomyelin to ceramide and phosphocholine [32] and is related not only to the regulation of the concentration of sphingomyelin in the cell membrane, but also to the aggregation of LDL, resulting in plaque formation in atherosclerosis [33]. In this study we confirmed that the activity of NSMase was inhibited by 27-hydroxycholesterol [34]. The inhibited activity of NSMase could result in an intracellular sphingomyelin accumulation. Our data does not support a previous report that 27-hydroxycholesterol enhanced phosphatidylcholine synthesis by activating CT in Chinese-hamster ovary cells [35].

In this study, we found that lovastatin and 27-hydroxycholesterol affected sphingomyelin content by different mechanisms. That is, lovastatin decreased sphingomyelin content by reducing phosphatidylcholine product, whereas 27-hydroxycholesterol increased sphingomyelin content by inhibiting NSMase. Therefore it is possible when the decreasing effect of lovastatin on sphingomyelin content was stronger than the stimulative effect of 27-hydroxycholesterol, a reduced sphingomyelin took place after lovastatin was added into the 27-hydroxycholesterol-treated cells.

To examine the possible relationship between the concentrations of sphingomyelin and cholesterol in the presence of lovastatin, we measured sphingomylin synthesis and cholesterol content after adding mevalonic acid and cholesterol, respectively, in the culture medium containing lovastatin. The results showed that the lovastatin-mediated decrease in cholesterol was completely reversed in the cells incubated with mevalonic acid or cholesterol, but diminished sphingomyelin was not reversed. Our findings indicate that the lovastatin-mediated decrease in sphingomyelin was not related to a diminished cholesterol concentration or synthesis. A similar result obtained from a clinic study showed that rosuvastatin dose-dependently lowered plasma sphingolipids and phospholipids and the decrease was independent of low-density lipoprotein lowering [36]. This study does not suggest a parallel association of the concentrations of sphingomyelin and cholesterol, although their parallel accumulation occurred in a variety of pathological conditions such as atherosclerosis and Niemann–Pick disease [37], [38]. Moreover, our findings that 27-hydroxycholesterol enhanced sphingomyelin content, but decreased cholesterol concentration in the cultured cells [6], [39] also indicate that their association did not always exist.

In summary, we have presented evidence in this study that (1) lovastatin induced a reduction of phosphatidylcholine concentration by inhibiting CT activity, resulting in a decrease of sphingomyelin content; and (2) the decreased sphingomyelin was not related to the diminished cholesterol concentration in the presence of lovastatin.

## Conflict of interest

The authors declare no conflict of interest.
